# Supplementary material for: Characteristics of Dutch ED patients and their journey through the acute care chain: A province-wide flash-mob study
Source: PLoS One. 2025 Apr 3;20(4):e0318510. doi: 10.1371/journal.pone.0318510 (PMC11967924; doi:10.1371/journal.pone.0318510)
Supplement: S1 Questionnaire — (DOCX) [file pone.0318510.s001.docx]

**Questionnaire 1**

Patient#:
Inclusion location:

| **Questionnaire patient TACC study – T**ime within the **A**cute **C**are **C**hain |
| --- |

| General information | |  |
| --- | --- | --- |
|  | **Sex** | Male / Female |
|  | **Age** | …… years |
|  | **Highest level of education** | No education – primary school -  Secondary school – vocational training – University |
|  | **Living situation** | Independent living – community dwelling with domiciliary care – assisted living – nursing home – other: ……… |
|  | **Room mates** | Yes / No |

| BEFORE YOUR VISIT AT THE EMERGENCY DEPARTMENT TODAY | | |
| --- | --- | --- |
|  | **Since what date have you been experiencing these symptoms for which you are now at the Emergency Room?** | …………………………………… |
|  | **In addition to the contact that brought you to the Emergency Room, have you had any previous contact with a healthcare provider in this disease episode?** | Yes / No |
|  | **When was the first contact with a healthcare provider during this disease episode?** | …………………………………… |
|  | **How many times have you had contact with a healthcare provider since your first contact mentioned in question 8?** | …………… times, including the first contact and your contact today. |
|  | **Has any medication been prescribed to you already?** | No / yes, date: …………………… |
|  | **What medication was prescribed?** | …………………………………… |

| YOUR VISIT AT THE EMERGENCY DEPARTMENT TODAY | | |
| --- | --- | --- |
|  | **What complaint or symptoms are you experiencing today?** | …………………………………… |
|  | **What time did you contact the referred healthcare provider today?** | Time: ……:…… h  No, I have had previous contact, but I could only get an appointment today. |
|  | **What time did you visit the general practitioner today, or did they come to your home?** | Time: ……:…… h |
|  | **What diagnosis did the doctor ultimately make at the Emergency Room?** | ………………………………………………………………………………. |

Patient#:
Inclusion location:

| **Questionnaire professional TACC study – T**ime within the **A**cute **C**are **C**hain  Questionnaire to be completed by the researcher   \|  \|  \| \|  \| \| --- \| --- \| --- \| --- \| \|  \| **Referred specialty** \| ……………………………………………………………………………… \| \| \|  \| **Referring healthcare provider** \| General practitioner – Nursing home doctor – hospital staff member - EMS- other……… \| \| \|  \| **Presenting complaint** \| ……………………………………………………………………… \| \| \|  \| **If the patient was referred via the GP what was the urgency level?** \| ……………………………………………………………………… \| \| \|  \| **Arrival time of ambulance at patient's location** \| Time: ……:…… h \| \| \|  \| **Ambulance urgeny level** \| ……………………………………………………………………………… \| \| \|  \| **Date + time of arrival from the ED** \| ……-……-…………  Time: ……:…… h \| \| \|  \| **Urgency level ED** \| ……………………………………………………………………………… \| \| \|  \| **Date + time of departure from the ED** \| ……-……-…………  Time: ……:…… h \| \| \|  \| **Diagnosis at the time of ED departure** \| ……………………………………………………………………………… \| \| \|  \| **Which examinations were conducted during the stay in the ED?** \| Bloodtest – ECG – X-ray – Ultrasound – CT-scan - Other……………………… \| \| \|  \| **Final specialty, to which specialty is the patient discharged?** \| ………………………………………………………………………………. \| \| \|  \| **If referring specialty ≠ final specialty and the patient is admitted, is it necessary to transfer the patient to another hospital?** \| Yes / No \| \| \|  \| **What happened after the ED?** \| Admission (go to question 15) - discharge (go to question 17) – first line care facility - other……………………… \| \| \|  \| **Which department was the patient admitted to?** \| Regular nursing ward - High care department such as an MC (Medium Care), Cardiac Care Unit (CCU), or Brain Care Unit - Intensive Care Unit (ICU) - Transfer to another hospital \| \| \|  \| **Date of discharge** \| ……-……-………… \| \| \|  \| **Is the patient still alive 7 days after presentation at the ER?** \| Yes (go to question 18) / No (go to question 19) \| \| \|  \| **Is the patient still alive 30 days after presentation at the ER?** \| Yes (go to question 19) / No (go to question 21) \| \| \|  \| **Date of death** \| ……-……-………… (ga naar vraag 20) \| \| \|  \| **Has the patient revisited the ED?** \| Yes (go to question 21) / No \| \| \|  \| **How many times has the patient revisited the ED after the initial presentation?** \| ……………………… time(s) \| \| |
| --- | --- | --- | --- | --- | --- | --- | --- | --- | --- | --- | --- | --- | --- | --- | --- | --- | --- | --- | --- | --- | --- | --- | --- | --- | --- | --- | --- | --- | --- | --- | --- | --- | --- | --- | --- | --- | --- | --- | --- | --- | --- | --- | --- | --- | --- | --- | --- | --- | --- | --- | --- | --- | --- | --- | --- | --- | --- | --- | --- | --- | --- | --- | --- | --- | --- | --- | --- | --- | --- | --- | --- | --- | --- | --- | --- | --- | --- | --- | --- | --- | --- | --- | --- | --- | --- | --- | --- | --- |
