# Supplementary material for: Characteristics of Dutch ED patients and their journey through the acute care chain: A province-wide flash-mob study
Source: PLoS One. 2025 Apr 3;20(4):e0318510. doi: 10.1371/journal.pone.0318510 (PMC11967924; doi:10.1371/journal.pone.0318510)
Supplement: S2 Table — Values are n(%) for ordinal variables and median (IQR) for continues variables, median (IQR). Abbreviations: ED – emergency department. *2 missing in eligible group **p<0.05. (DOCX) [file pone.0318510.s002.docx]

# **Supplement 2**

**Table 1 – Comparison of patients with and without prespecified complex conditions (n=583)**

|  | **Included patients**  **(n = 583)** | **Eligible, non-included, patients (n = 211)** | **p-value** |
| --- | --- | --- | --- |
|  | n (%) or median (IQR) | n (%) or median (IQR) |  |
| **Sex**, n (%) *  Male  Female  Missing | 293 (50.3%)  290 (49.7%) | 109 (51.7%)  100 (47.4%)  2 (0.9%) | 0.638 |
| **Age** **in years**, median (IQR) | 65 (47-76) | 65 (44-79) | 0.509 |
| **Urgency level ED**, n (%)  Highly urgent  Urgent  Unknown | 103 (17.5%)  474 (80.9%)  9 (1.6%) | 52 (24.7%)  148 (70.1%)  11 (5.2%) | **0.013**** |
| Values are n(%) for ordinal variables and median (IQR) for continues variables, median (IQR) Abbreviations: ED – emergency department. *2 missing in eligible group ****p<0.05.** | | | |
